# Potsdam Eye-Movement Corpus for Scene Memorization and Search With Color and Spatial-Frequency Filtering

**DOI:** 10.3389/fpsyg.2022.850482

**Published:** 2022-02-23

**Authors:** Anke Cajar, Ralf Engbert, Jochen Laubrock

**Affiliations:** ^1^Department of Psychology and Research Focus Cognitive Sciences, University of Potsdam, Potsdam, Germany; ^2^Medizinische Hochschule Brandenburg Theodor Fontane, Neuruppin, Germany

**Keywords:** eye movements, corpus dataset, scene viewing, object search, scene memorization, spatial frequencies, color, central and peripheral vision

## 1. Introduction

Corpus-based analyses of eye movements represent a powerful approach to further theories on free-viewing, memorization, and search behavior in real-world scenes; for example, when assessing the roles of low-level visual processing and top-down control for eye guidance (Schütt et al., 2019). However, only few large eye-movement corpora for scene viewing are publicly available (e.g., Judd et al., 2009; Borji and Itti, 2015; Wilming et al., 2017).

The importance of eye-movement corpora has increased with the turn to scan-path analyses of time-ordered fixations (Engbert et al., 2015), which require many fixations per scene. For example, the application of spatial statistics and point process theory to eye-movement data (e.g., Barthelme et al., 2013) allows for investigating individual differences in spatial correlations between fixation locations (Trukenbrod and Engbert, 2012). In mathematical models of eye-movement control during scene viewing, sequential likelihood methods (Schütt et al., 2017), which are based on the availability of time-ordered fixation sequences, can achieve highest precision in the evaluation of theoretical models (Engbert et al., 2022). As a result, individual viewing behavior can be predicted in a fully Bayesian dynamical modeling framework (Schwetlick et al., 2020). Therefore, we expect that the availability of large eye-movement corpora will become a major resource for advancing theoretical models of eye-movement control and visual cognition.

The present paper presents a database with a large new eye-movement corpus for scene viewing under different task instructions and experimental conditions. A total of 200 participants were instructed to search for an object in 120 real-world scenes or to memorize 90 different scenes while their eye movements were recorded. Half of the participants saw scenes in color, the other half saw the same scenes in grayscale. All participants were exposed to the same viewing conditions while performing the tasks: During scene memorization and object search, spatial-frequency filters aligned with the viewer's gaze in real-time were applied to either central vision (from the point of fixation up to 5° retinal eccentricity) or peripheral vision (>5° eccentricity). The filters attenuated either low spatial frequencies in the scenes, which carry coarse-scale information about the general shape and structure of objects, or high spatial frequencies, which carry fine-scale information about sharp edges and surface textural properties. The four experimental conditions (i.e., central or peripheral low-pass filtering and central or peripheral high-pass filtering) were contrasted with a control condition without any filtering.

Thus, our database provides eye-movement data from many participants who inspected color or grayscale scenes under different task instructions while visual-cognitive processing difficulty was manipulated. In addition to (i) preprocessed eye-movement data, the corpus provides (ii) the full set of 210 images presented as stimuli for the experiments, (iii) object annotations for all scenes, with object labels and coordinates of manually drawn polygons around objects, and (iv) heatmaps and cluster identifications based on empirical fixation densities on the scenes. Note that statistical analyses for the search task regarding the effects of spatial-frequency and color filtering on eye-movement and search behavior have already been reported in Cajar et al. (2020).

We hope that the provided materials and data will be used in various ways. The scenes might serve as stimuli in new experiments. Eye-movement data can be used for analyses of task differences (scene memorization vs. search) and the importance of color and spatial frequencies while performing these tasks; as spatial frequencies are attenuated gaze-contingently in central or peripheral vision, the contributions of central and peripheral vision to scene processing can also be investigated. Furthermore, the data can be used for validating computational models of attention and eye-movement control during scene viewing; and finally, the corpus data allow time-dependent analyses and analyses on an object- or cluster-based level.

## 2. Stimulus Material

Stimulus images presented during the experiment were landscape-format photographs of real-world scenes with a resolution of 1024 × 768 pixels, subtending corresponding visual angles of 38.2° (horizontally) and 28.6° (vertically). Stimulus images in the memorization task were 90 outdoor photographs mostly depicting leisure-time scenes. Scene images for the visual search task were 120 photographs of real-world scenes from the BOiS database (Mohr et al., 2016), comprised of 100 photographs depicting indoor and 20 photographs depicting outdoor scenes. Original BOiS scenes were cropped and downsized for the present experiment.

### 2.1. Object Annotations

**Figure 1:**
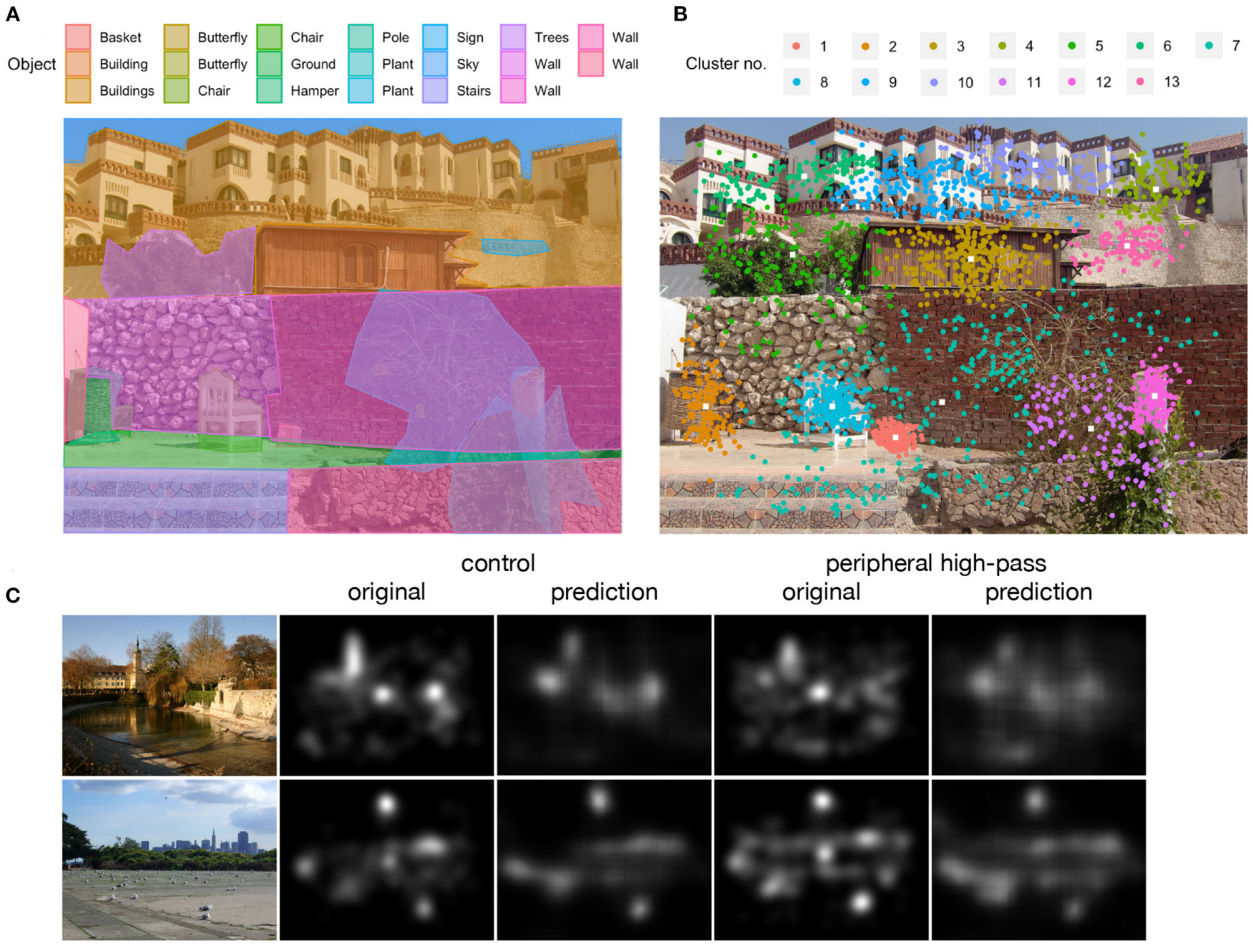
Illustration of object annotations, fixation clusters, and heatmaps for scenes from the memorization task. **(A)** Scene with manually drawn polygons outlining individual objects or object groups with corresponding object labels. **(B)** Fixation clusters based on empirical fixation positions across all participants and spatial-frequency filter conditions on the color version of the scene. **(C)** Exemplary heatmaps of fixation distributions for the control condition (no filtering) and peripheral high-pass filtering based on empirical fixation positions across all participants and color conditions (*original*) and on predictions of a convolutional neural network model for that fixation distribution (*prediction*). White color indicates the highest and black color indicates the lowest density of fixations.

Objects in all 210 scenes were identified and annotated manually using an editor developed for the annotation of objects in comics (Dunst et al., 2017). Using this editor, polygons were drawn manually either around individual objects or around groups of objects if objects were located in the scene background (e.g., a group of trees). Each object or object group was then tagged with a descriptive label. For the scenes used in the search task, the target object was additionally labeled as target. For an example scene with object annotations see Figure 1A. In the database, object annotations are provided as individual text files for each scene containing the object labels together with the x- and y-coordinates of the polygons outlining the objects. Since research indicates that attention in scene viewing is driven strongly by objects (e.g., Nuthmann and Henderson, 2010), object annotations can be used to map participants' empirical fixation positions onto scene objects and perform object-based eye-movement analyses.

## 3. Experimental Details

### 3.1. Participants

Two hundred people (38 male, mean age: 22.5 years, range: 16 to 40 years) who were students at the University of Potsdam or pupils at local schools participated in the experiment. Participants had normal vision or corrected-to-normal vision (via glasses or contact lenses) and normal color discrimination. Color discrimination was assessed using chromaticity diagrams from Velhagen and Broschmann (2003). Additional information for each participant (e.g., visual acuity, dominant eye, measures for verbal and performance IQ) is provided in the database. All subjects were naive with respect to the scientific questions underlying the experiment and received course credit or monetary compensation for participation.

### 3.2. Apparatus

Participants were seated in a quiet, dimly lit room opposite to the operator desk, each one facing their own monitor so that participant and operator could not see each other. Participants were positioned at a viewing distance of 60 cm (23.6 inches) from the monitor with their head stabilized by a head-chin rest. Scenes were presented on an Iiyama VisionMasterPro 514 monitor with a resolution of 1024 × 768 pixels and a refresh rate of 150 Hz. During the eye-tracking experiment stimuli and response collection were controlled with MATLAB (version R2015b; The Mathworks, Natick, MA) using the Psychophysics Toolbox (PTB-3; Brainard, 1997; Kleiner et al., 2007), which includes the Eyelink Toolbox Cornelissen et al. (2002). Gaze position of the dominant eye was tracked during binocular viewing with an EyeLink 1000 Tower Mount system (version 4.56; SR Research, Ontario, Canada). System latency from eye movement to screen update (as a sum of frame duration, tracker latency, and eye-velocity computation) was smaller than or equal to 9.5 ms.

### 3.3. Gaze-Contingent Spatial-Frequency Filtering

Spatial-frequency filtering was realized in the Fourier domain with Gaussian filters with cutoff frequencies of 1 cpd (= cycles per degree of visual angle) for low-pass filters and 9 cpd for high-pass filters. Thus, low-pass filtering attenuated spatial frequencies above 1 cpd and high-pass filtering attenuated spatial frequencies below 9 cpd. For gaze-contingent filtering in central or peripheral vision, filters were applied either inside or outside a moving window, which was centered on the current gaze position in real-time. The radius of the window was 5°, thus roughly dividing central from peripheral vision (cf. Larson and Loschky, 2009). For details on the specific spatial-frequency filters used in the experiment and technical details on how these filters were applied gaze-contingently to the central or peripheral visual field see Cajar et al., 2020.

### 3.4. Design

**Figure 2:**
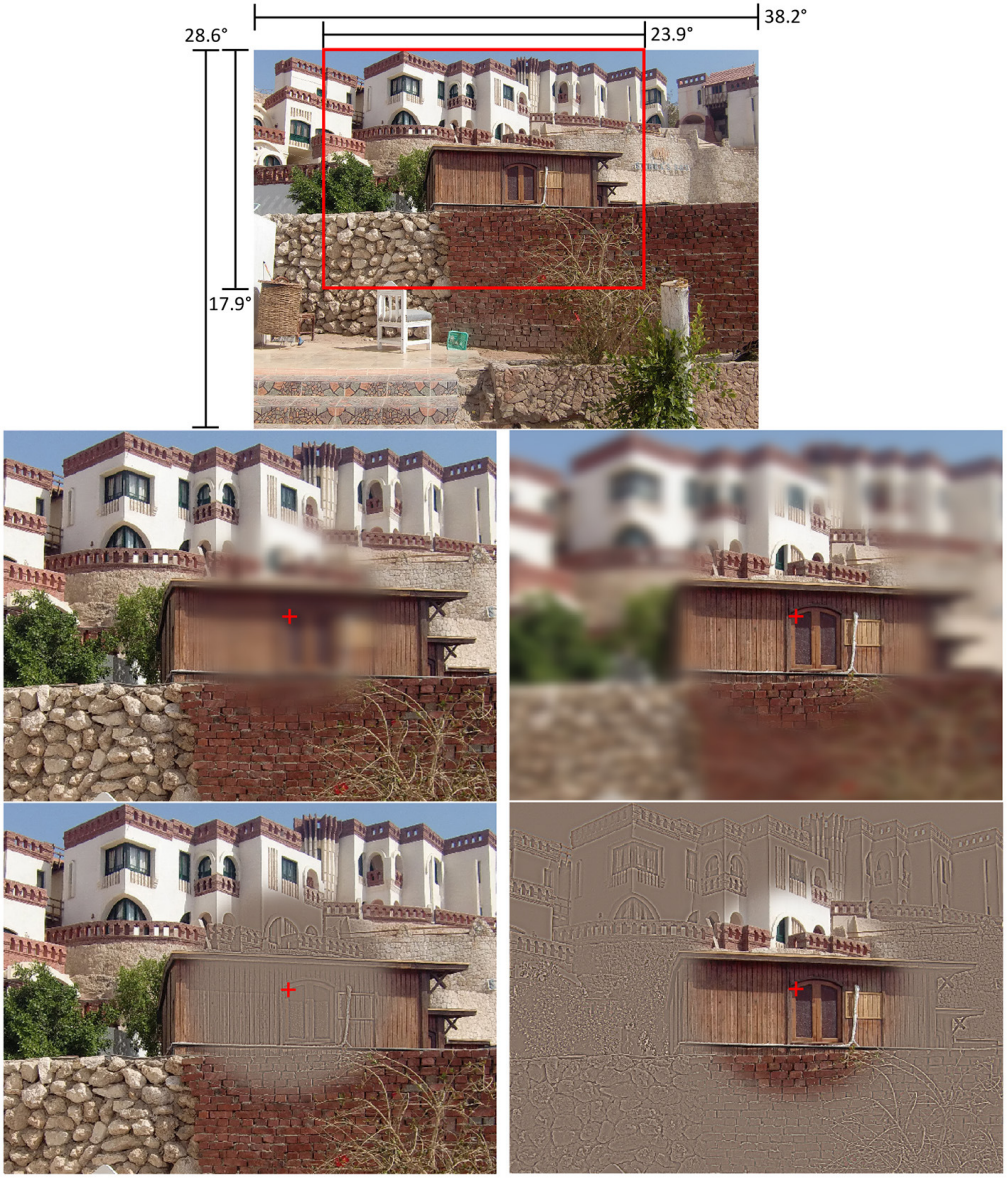
Illustration of the five filter conditions. The red cross indicates the current gaze position. The top panel shows the original stimulus from the scene memorization task in the unfiltered control condition. Below, the four filter conditions are illustrated with cropped and zoomed-in versions of the original stimulus (indicated by the red frame) to better illustrate the filter effects. (Middle row, left) Central low-pass filter. (Middle row, right) Peripheral low-pass filter. (Bottom row, left) Central high-pass filter. (Bottom row, right) Peripheral high-pass filter.

Scenes were either presented without any filtering (control condition) or with one of four spatial-frequency filters: central low-pass, central high-pass, peripheral low-pass, or peripheral high-pass (for example stimuli, see Figure 2). As a result, there were 18 trials per condition for the memorization task and 24 trials per condition for the search task. With the search task, half of the trials were target absent and the other half were target present trials; of the latter, half of the trials presented the target at a predictable and the other half at an unpredictable location regarding scene context (e.g., a remote-control lying next to the television set vs. placed on the living room floor). In both memorization and search tasks, scenes were presented in their original color version to half of the participants and in a grayscale version to the other half of the participants.

A Latin square design assured counterbalancing of filter condition–scene(–target location) assignments across participants. Scenes and filter conditions were presented in random order. Half of the participants started with the memorization-task session, the other half started with the search-task session.

### 3.5. Procedure

In both sessions of the experiment each trial started with a fixation check, where the eyes had to fixate on a black cross in the center of the screen and stay inside an invisible rectangle of size 1.5° × 1.5° for 150 ms. If the fixation check was successful, image presentation started. For scene memorization, each scene was presented for 7 s. Participants were instructed to explore scenes carefully in preparation for questions regarding scene content. Questions with three response alternatives that participants could answer with the computer mouse were presented after a randomly chosen 50% of the scenes. Questions were comparably difficult, with an answer accuracy of about 75% (see Cajar et al., 2016).

With the object-in-scene search task, each trial started with a picture cue of the target that was shown for 2 s after the initial fixation check. Before the scene was revealed, participants had to fixate a black cross in the center of the screen to ensure they always started searching in the scene center. During search, participants had to decide as quickly as possible whether the target object was present or absent in the scene and indicate their decision by pressing either the left or right button on the computer mouse. After a decision was made, the scene was offset immediately. Mean search time across all experimental conditions was 3.8 s (*SD* = 2.7 s).

## 4. Eye-Movement Data

Data were collected over a period of 21 months from July 2017 to March 2019. In the introduction, we touched a number of scientific questions that can be investigated using these data, some of which have received little attention so far. First, the influence of different task instructions on eye-movement behavior can be analyzed (i.e., scene memorization vs. scene search; see also Castelhano et al., 2009; Mills et al., 2011; Henderson et al., 2013). Second, the importance of color for scene processing can be investigated (see also Nuthmann and Malcolm, 2016; Cajar et al., 2020). Third and forth, the data can be used to expand knowledge on the individual contributions of central and peripheral vision to scene viewing and on the processing of different spatial-frequency bands in central and peripheral vision (see, e.g., van Diepen and Wampers, 1998; Loschky et al., 2005; Nuthmann, 2014; Cajar et al., 2016).

### 4.1. Data Preparation

Saccades and fixations were detected from raw time series of gaze positions using a velocity-based algorithm (Engbert and Kliegl, 2003; Engbert and Mergenthaler, 2006) with a relative velocity threshold of 6 median-based standard deviations and a minimum duration of 8 data samples. From 42,000 trials recorded, 124 trials (or 0.69%) in the memorization task and 68 trials (or 0.28%) in the search task were removed owing to poor calibration or substantial data loss. The latter was given when more than four eye blinks occurred during a single trial or when the pupil could not be tracked properly during a large part of the trial owing to poor recording. Single fixations and saccades were removed, if they were adjacent to eye blinks or were outside the monitor area. If the first or last trial event was an ongoing saccade, it was discarded as well. We provide the preprocessed eye-movement data for the 200 participants of the corpus experiment on a fixation-by-fixation basis.

### 4.2. Cluster Identification

Empirical fixation positions from all participants were grouped into clusters for each color and grayscale scene. To determine clusters, we used the R package *mclust* for model-based clustering (Scrucca et al., 2016). *mclust* models the data based on Gaussian finite mixture models with different covariance structures and different numbers of mixture components. For parameter estimation, an expectation-maximization algorithm is used. The best model for describing the empirical data, with the best number of mixture components and covariance parameters, was selected using the Bayesian Information Criterion. We computed model-based clusters by assuming different shapes, volumes, and orientations for fixation clusters in each scene and a maximum number of 20 mixture components or clusters (note, however, that results did not change qualitatively by assuming a maximum of 70 components).

We provide the R script for cluster identification with *mclust* and the resulting cluster information in various forms. First, the database contains text files with only the cluster information for each scene, that is, the cluster numbers and x- and y-coordinates (in screen coordinates) of the respective cluster centers. Second, we provide the trial and eye-movement data with cluster information appended, that is, the trial and fixation information for each fixation in the experiment and the aforementioned cluster information. Third, figures of the scenes with clusters plotted on top of them are provided (see Figure 1B). Using this cluster information one can, for example, compute refixation or transition probabilities between clusters depending on spatial-frequency filtering conditions (and also compare those between tasks and color/grayscale scenes).

### 4.3. Heatmaps

Heatmaps can be used to visually compare fixation distributions between the experimental conditions, but also as learning objectives for fixation location predictions, for example, in training a convolutional neural network. Heatmaps were created for fixations of all participants in a given presentation condition (i.e., color or grayscale scene; central or peripheral filter; high-pass filter, low-pass filter, or no filter). Fixation locations were convolved with a two-dimensional Gaussian filter with a bandwidth of 27 pixels (corresponding to 1.0 degree of visual angle) using the *kdeplot* function from the *Seaborn* package in Python (Waskom, 2021). Figure 1C shows exemplary heatmaps for the control condition compared with peripheral high-pass filtering. White color indicates the highest density of fixations and black color indicates the lowest density. The figure indicates that empirical fixation distributions became less focused when peripheral high-pass filters made object selection in the periphery more difficult. This is also evident in the predictions of a convolutional neural network model trained to predict where people attend on other images (Laubrock and Dubray, 2019).

### 4.4. Statistical Analyses of Eye-Movement Behavior During Scene Search

R scripts with statistical analyses and figures for search performance and eye-movement behavior in the search task of the experiment, as reported in Cajar et al. (2020), can be found in Open Science Framework under project “How spatial frequencies and color drive object search in real-world scenes,” https://osf.io/jq56s/ (doi:10.17605/OSF.IO/JQ56S).

## 5. Database Details

All material described in the present paper is publicly available via Open Science Framework under project “Potsdam eye-movement corpus for scene memorization and search with color and spatial-frequency filtering,” https://osf.io/8cwt6/ (doi:10.17605/OSF.IO/8CWT6). It can be downloaded as a single archive (about 3.3 GB). Alternatively, files or folders can be downloaded individually.

The repository contains separate folders for the memorization and the search task of the experiment, named “Memorization” and “Search” respectively. The subfolders “Stimuli” contain the scenes used as stimuli in the eye-tracking experiment, that is, the 90 scenes used for the memorization task and the cropped and resized versions of the 120 scenes from the BOiS database (Mohr et al., 2016) used for the search task. The names of the search scenes correspond to the original names of the stimuli in the BOiS database. For each scene, the original color version and the grayscale version is provided (folders “Color” and “Grayscale” respectively). The preprocessed eye-movement data on fixation-by-fixation basis for the memory and search sessions are stored as individual text files named “Potsdam_Scene_Memorization_Corpus.dat” and “Potsdam_Scene_Search_Corpus.dat,” respectively. Text files in XML format containing object identifications and annotations for the scenes can be found in the folder “Object annotations.” Heatmaps are stored in the folder “Heatmaps,” with subfolders for the five different spatial-frequency filtering conditions for color and grayscale scenes. Scene clusters can be found in the folder “Empirical fixation clusters,” with individual text files for cluster information and cluster plus trial and fixation information for color and grayscale scenes. The subfolder “Scenes with clusters” contains the scenes with the clusters plotted onto them, separated by color and grayscale scenes. Information about participants can be found in the file “Participant_information.dat” in the main folder of the database.

## Data Availability Statement

The datasets presented in this study can be found in online repositories. The names of the repository/repositories and accession number(s) can be found at: Potsdam eye-movement corpus for scene memorization and search with color and spatial-frequency filtering, https://osf.io/8cwt6/.

## Ethics Statement

Ethical review and approval was not required for the study on human participants in accordance with the local legislation and institutional requirements. Written informed consent from the participants' legal guardian/next of kin was not required to participate in this study in accordance with the national legislation and the institutional requirements.

## Author Contributions

All authors developed the concept for the database and designed the eye-tracking experiment. All authors contributed photos for the memorization session of the experiment. AC modified photos for the search session of the experiment. Student assistants and AC generated object annotations for the scenes. JL provided CNN features for the heatmaps. AC programmed the experiment, provided cluster analyses, as well as the statistical analyses for the search task, created and managed the OSF repository, and wrote the manuscript. JL and RE revised the manuscript.

## Funding

This work was funded by Deutsche Forschungsgemeinschaft (DFG) *via* grants LA 2884/1 to JL and EN 471/10 to RE.

## Conflict of Interest

The authors declare that the research was conducted in the absence of any commercial or financial relationships that could be construed as a potential conflict of interest.

## Publisher's Note

All claims expressed in this article are solely those of the authors and do not necessarily represent those of their affiliated organizations, or those of the publisher, the editors and the reviewers. Any product that may be evaluated in this article, or claim that may be made by its manufacturer, is not guaranteed or endorsed by the publisher.
